# Anti-Neuroinflammatory Effects of a Representative Low-Molecular-Weight Component Isolated from *Codium fragile* Through Inhibition of the NF-κB Pathway in Microglia and Macrophage Cells

**DOI:** 10.3390/md24010038

**Published:** 2026-01-13

**Authors:** Gyoyoung Lee, Yezhi Jin, Seul Ah Lee, Sook-Young Lee, Hwan Lee, Zisheng Nan, Chi-Su Yoon, Dong-Sung Lee

**Affiliations:** 1 Research Institute of Pharmaceutical Sciences (RIPS), College of Pharmacy, Chosun University, Gwangju 61452, Republic of Korea; dlrydud612@naver.com (G.L.); yezhi102@gmail.com (Y.J.); ghksdldi123@hanmail.net (H.L.); zisheng1125@gmail.com (Z.N.); 2 Department of Oral Biochemistry, College of Dentistry, Chosun University, Gwangju 61452, Republic of Korea; seulah21@naver.com; 3 Marine Healthcare Research and Evaluation Center, Chosun University, Wando 59146, Republic of Korea; seedbank@chosun.ac.kr; 4 Institute of Pharmaceutical Research and Development, College of Pharmacy, Wonkwang University, Iksan 54538, Republic of Korea

**Keywords:** *Codium fragile*, inflammation, NF-κB, microglia, macrophage

## Abstract

The worldwide incidence of neurodegenerative diseases (ND), such as dementia, has increased, and neuroinflammation is considered a crucial factor in the development of ND. *Codium fragile* is considered ocean waste in many countries; however, some countries, including Korea, consume it as a food resource. In this study, a major low-molecular-weight component and chemical marker, uracil, was isolated from the aqueous extracts of *C. fragile* (AECF); additionally, its content was measured through HPLC quantitative analysis. AECF and uracil were examined for their anti-inflammatory activities against lipopolysaccharide (LPS)-stimulated BV2 microglia and RAW264.7 macrophage cell lines under inflammation conditions. The results showed that AECF and uracil inhibited the production of pro-inflammatory cytokines by suppressing the NF-κB pathway.

## 1. Introduction

Seaweed represents a remarkably diverse group of organisms classified into three primary categories: Rhodophyta (red), Chlorophyta (green), and Ochrophyta (brown) [1]. Their ecological significance extends beyond their mere presence, as they function as ecosystem engineers by providing critical habitat structures and supporting complex marine communities. The use of seaweed as food has deep historical roots in coastal populations worldwide, with approximately 600 species being consumed globally. Growing consumer interest in nutritional quality and health-conscious dietary choices has sparked renewed attention to seaweed as a source of functional foods [2]. Seaweeds have been consumed for a long time, mainly in Asian countries, such as Japan, China, and Korea [3]. Among them, *Codium fragile* is recognized as marine waste in some countries [4], but it is used as food in Korea and has a high market value. *C. fragile* is a green seaweed belonging to the family Codiaceae. It ranges from 10 to 40 cm in height and includes repeatedly branching cylindrical segments [5]. The *Codium* green algae species are known to contain metabolites such as sulfated polysaccharides in their cell walls [6,7]. The anti-inflammatory, anti-obesity, and anti-diabetic effects of *C. fragile* have been previously reported [8,9,10,11,12]. Lee et al. [13] reported that the aqueous extract of *C. fragile* (AECF) exerts potential anti-inflammatory effects by suppressing NF-κB (nuclear factor-kappa B) activation and MAPK (mitogen-activated protein kinase) pathways in vitro, as well as inhibiting carrageenan-induced rat paw edema thickness in vivo. However, its anti-neuroinflammatory effects in BV2 microglia cells have not been studied yet. Uracil (pyrimidine-2,4(1H,3H)-dione) is a naturally occurring member of the pyrimidine family and an active component of ribonucleic acid (RNA) [14]. In 1900, uracil was initially isolated from the hydrolytic products of yeast nucleic acids [15]. Several research findings indicate that uracil and its derivatives have effects on treating viral infections, cancer, diabetes, thyroid disorders, and autosomal recessive disorders [16,17]. However, the biological effects of uracil on microglia-induced inflammation have not yet been evaluated.

As the global population ages, the number of individuals affected by brain diseases, such as dementia, continues to increase steadily, prompting extensive research efforts to develop effective treatments. Neuroinflammation is a major contributing factor to the development of brain diseases, and numerous natural products are being investigated for their therapeutic potential in addressing this condition [18,19,20,21,22]. Neuroinflammation refers to the inflammatory responses occurring in the brain. Although macrophages typically mediate inflammatory responses, microglia primarily regulate these responses. Microglia exist in two phenotypes, M1 and M2, and, upon exposure to inflammatory stimuli, they secrete various pro-inflammatory cytokines, leading to their transformation into a chronic inflammatory state [23]. The functions of microglia are regulated by inhibitory cytokines. Transforming growth factor-β (TGF-β) inhibited nitric oxide (NO), tumor necrosis factor-α (TNF-α) production, and the expression of the IL-6 receptor by lipopolysaccharide (LPS)-stimulated microglia [24,25]. This characteristic is similar to that of macrophages; therefore, microglia are recognized as a type of macrophage in the brain [26]. Interleukin-10 (IL-10) is a key anti-inflammatory cytokine that counterbalances excessive inflammatory responses. In macrophages, LPS stimulation can induce IL-10 as a negative-feedback response, and several anti-inflammatory modulators further augment IL-10 production. In contrast, IL-10 responses to LPS in microglia are more context-dependent, and decreases in IL-10 have also been reported in LPS-stimulated BV2 microglia [27,28,29,30,31,32,33]. Accordingly, we evaluated the anti-neuroinflammatory activity of microglia while simultaneously validating their anti-inflammatory activity in macrophages. In this study, uracil was isolated from AECF and recognized as a major low-molecular-weight component and chemical marker of the extract by HPLC analysis. Therefore, the anti-inflammatory effects of AECF, along with uracil, were examined in RAW264.7 macrophages and BV2 microglia cells.

## 2. Results

### 2.1. Isolation and Identification of the Representative Low-Molecular-Weight Component Isolated from C. fragile

**Figure 1:**
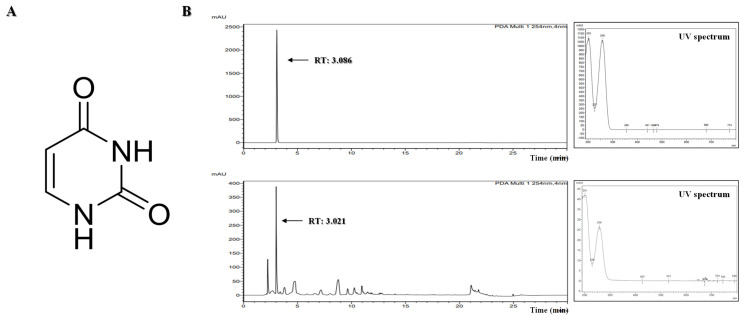
Chemical structure of uracil (**A**) and HPLC chromatograms of uracil and AECF (**B**).

The AECF was dissolved in water and sequentially partitioned with dichloromethane, ethyl acetate, and n-butanol to yield four solvent fractions. HPLC analysis revealed a major marker compound in the n-butanol fraction. This fraction was subjected to open-column chromatography using a YMC GEL ODS-A column with a gradient elution of 10–100% methanol in water, yielding the CG-B10 subfraction (479 mg). CG-B10 was further purified by Sephadex LH-20 column chromatography using 20% methanol as the mobile phase, yielding the CG-B10-S5 fraction. Preparative HPLC was performed, and the peak elution at 9.2 min was collected to yield 3.5 mg of CG-B10-S5-1. The structure of CG-B10-S5-1 was elucidated with the ^1^H-NMR and ^13^C-NMR spectrum in DMSO-d_6_, and its molecular weight was confirmed by high-resolution mass spectrometry (HR-MS), showing *m*/*z* 113.0341 [M + H]^+^ in positive ion mode and *m*/*z* 111.0202 [M − H]^−^ in negative ion mode (Figures S2–S5). Based on spectroscopic data, the compound was identified as uracil. The chemical structure of uracil is shown in Figure 1A. Furthermore, uracil was quantitatively analyzed in an AECF to evaluate its potential as a chemical marker. The uracil content was measured at concentrations of 5, 7.5, and 10 mg/mL using HPLC, and the results were consistent across replicates. The average uracil content was calculated to be 2.2900 ± 0.0389 mg/g of dried extract, which corresponds to 0.2290 ± 0.0039% of the dried sample (Figure S1).

### 2.2. Inhibitory Effects of AECF and Uracil on Nitric Oxide (NO) Production in LPS-Stimulated RAW264.7 and BV2 Microglia Cells

**Figure 2:**
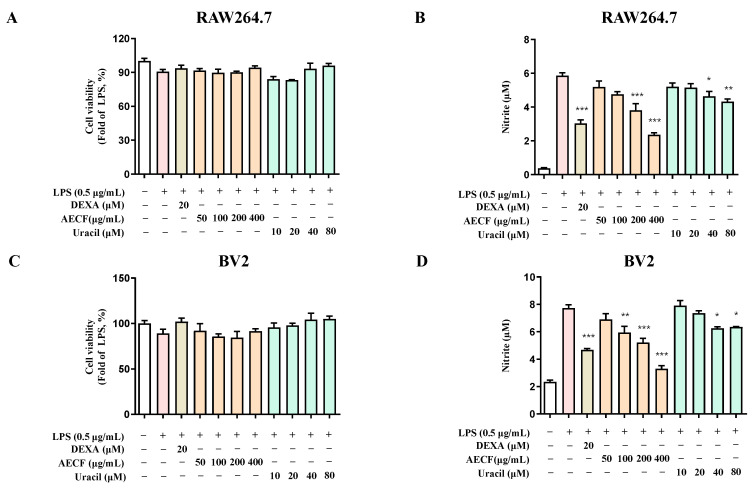
AECF and uracil effects on cell viability and the inhibitory effect on the nitrite production in RAW264.7 and BV2 microglia cells. Cell viability of RAW264.7 (**A**) and BV2 (**C**) cells was incubated for 24 h with 50–400 μg/mL of AECF or 10–80 μM of uracil. Cell viability was determined using 3-(4,5-dimethylthiazol-2-yl)-2,5-diphenyltetrazolium bromide assays. The inhibitory effects of AECF and uracil on nitrite production were evaluated in RAW264.7 (**B**) and BV2 (**D**) cells. Cells were pre-treated with various concentrations of AECF and uracil for 3 h, followed by stimulation with LPS. Date is expressed as the mean ± SD value of 3 independent experiments. * *p* < 0.05, ** *p* < 0.01, and *** *p* < 0.001 vs. LPS. Dexamethasone (DEXA) was used as the positive control. White bars indicate untreated control cells; pink bars indicate LPS-stimulated cells; light orange bars represent dexamethasone (DEXA)-treated cells; orange bars represent AECF-treated groups; and green bars represent uracil-treated groups.

Pro-inflammatory cytokines play a crucial role in the immune response of brain-resident immune cells. Previous studies have reported that lipopolysaccharide (LPS) induces the activation of NF-κB, which subsequently increases the production of interleukin (IL)-6 and NO [34]. Based on this, the effects of AECF and the isolated compound uracil on NO production were investigated in LPS-stimulated RAW264.7 macrophages and BV2 microglial cells. Briefly, RAW264.7 and BV2 microglia cells were pretreated with various concentrations of the extract and uracil for 3 h, followed by stimulation with LPS for 24 h. The results showed that, in RAW264.7 cells, AECF at (200 and 400 μg/mL) and the uracil compound at (40 and 80 μM) significantly inhibited NO production. In BV2 microglia cells, the AECF at concentrations ranging from 50 to 400 μg/mL and the uracil compound at 40 and 80 μM also significantly suppressed NO production (Figure 2). These findings suggest that uracil isolated from *C. fragile* may be a potential candidate for alleviating neuroinflammation.

### 2.3. Inhibitory Effects of AECF and Uracil on IL-6, Tumor Necrosis Factor (TNF)-α, and Interleukin-10 (IL-10) Production in LPS-Stimulated RAW264.7 and BV2 Microglia Cells

**Figure 3:**
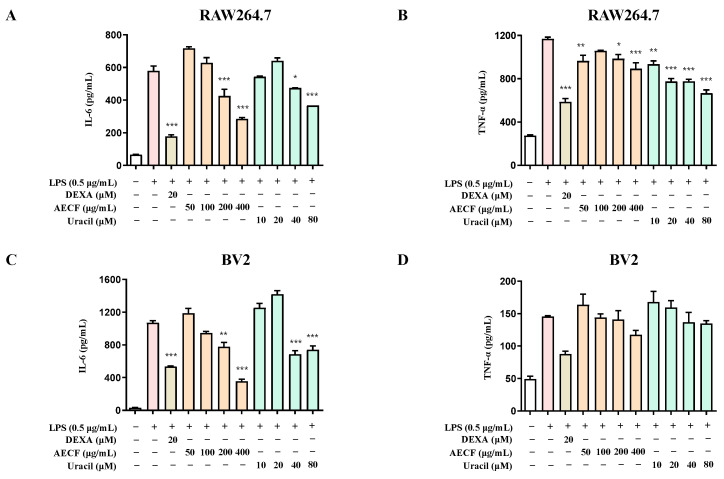
Inhibitory effects of inflammatory cytokines IL-6 and TNF-α on AECF and uracil in RAW264.7 macrophages and BV2 microglia. The inhibitory effects of IL-6 production in RAW264.7 (**A**) and BV2 (**C**) cells was induced for 3 h by pre-treatment with 50–400 μg/mL of AECF or 10–80 μM of uracil, followed by treatment with LPS. The inhibitory effects of TNF-α production in RAW264.7 (**B**) and BV2 (**D**) cells was induced for 24 h by pre-treatment with 50–400 μg/mL of AECF or 10–80 μM of uracil, followed by treatment with LPS. Data are presented as mean ± SD (*n* ≥ 3), and statistical significance is indicated as * *p* < 0.05, ** *p* < 0.01, and *** *p* < 0.001 versus LPS-treated control. Dexamethasone (DEXA) was used as the positive control. White bars indicate untreated control cells; pink bars indicate LPS-stimulated cells; light orange bars represent dexamethasone (DEXA)-treated cells; orange bars represent AECF-treated groups; and green bars represent uracil-treated groups.

**Figure 4:**
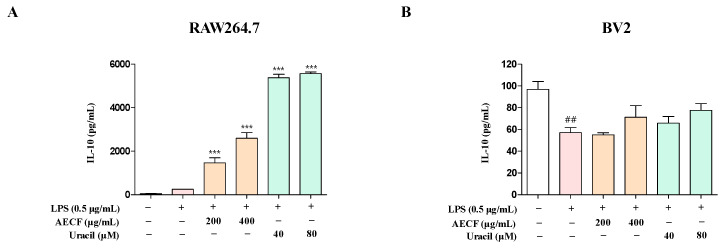
Effects of AECF and uracil on IL-10 production in RAW264.7 macrophages and BV2 microglia cells. IL-10 production in RAW264.7 (**A**) and BV2 (**B**) cells was induced by pre-treatment with AECF (200 and 400 μg/mL) or uracil (40 and 80 μM) for 3 h, followed by LPS stimulation. All data are presented as the mean ± SD (*n* ≥ 3), and statistical significance is indicated as *** *p* < 0.001 compared with the LPS-treated control and ^##^ *p* < 0.01 compared with the non-treated control. White bars indicate untreated control cells; pink bars indicate LPS-stimulated cells; orange bars represent AECF-treated groups; and green bars represent uracil-treated groups.

The effects of AECF and uracil on IL-6 and TNF-α production were evaluated in LPS-stimulated RAW264.7 and BV2 microglia cells. Cells were pretreated with various concentrations of the extract and uracil for 3 h, followed by stimulation with LPS for 24 h. ELISA analysis showed that, in RAW264.7 cells, both the extract (200 and 400 μg/mL) and uracil (40 and 80 μM) significantly inhibited IL-6 production. In BV2 microglia cells, IL-6 levels were significantly reduced at the same concentrations. However, in BV2 microglia cells, TNF-α production was not suppressed by either the extract or uracil treatment (Figure 3). To evaluate the immunoregulatory effects of AECF and uracil, IL-10 levels were measured in LPS-stimulated RAW264.7 macrophages and BV2 microglia. In RAW264.7 macrophages, LPS stimulation slightly increased the IL-10 compared with the untreated control, and co-treatment with AECF (200, 400 μg/mL) or uracil (40, 80 μM) further increased IL-10 levels in a concentration-dependent manner (Figure 4A). In BV2 microglia, LPS significantly decreased the IL-10 relative to the control group, whereas co-treatment with AECF or uracil showed a restorative upward trend toward the baseline; however, the difference versus the LPS-treated group did not reach statistical significance (Figure 4B). These findings suggest that uracil isolated from *C. fragile* may be a potential candidate for modulating neuroinflammatory responses.

### 2.4. Inhibitory Effect of C. fragile Extract and Uracil on iNOS and COX-2 Protein Expression in RAW 264.7 and BV2 Microglia Cells

**Figure 5:**
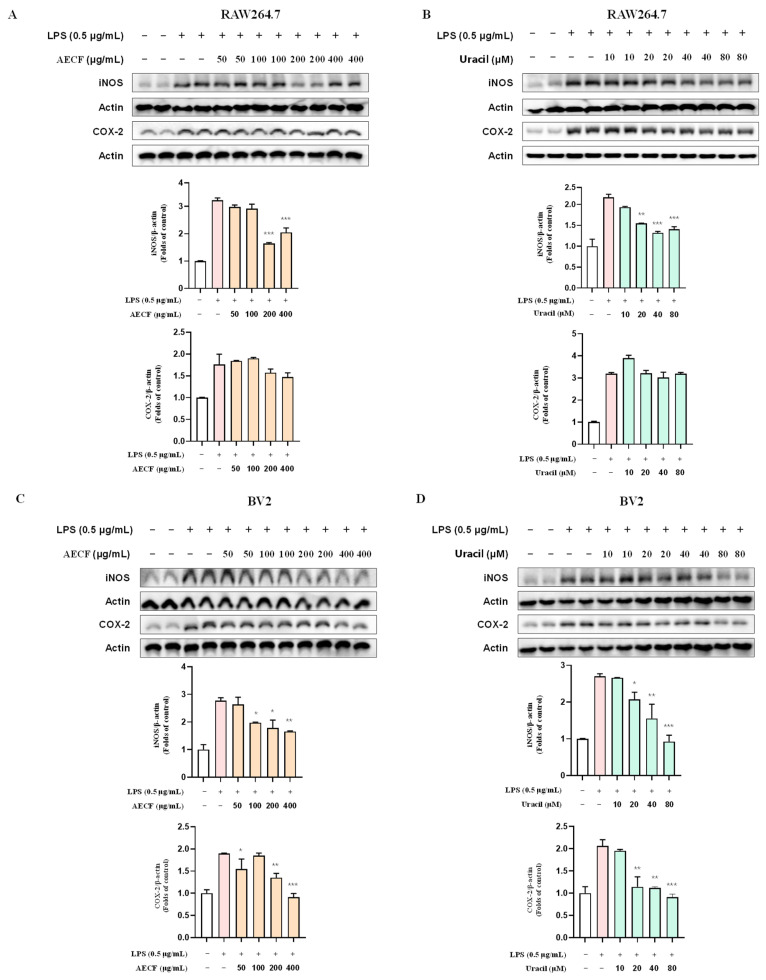
Western blot analysis of iNOS and COX-2 expression in RAW264.7 and BV2 microglia cells treated with AECF or uracil was performed, where RAW264.7 macrophages were stimulated with LPS (0.5 μg/mL) and subsequently treated with AECF (50–400 μg/mL) (**A**) or uracil (10–80 μM) (**B**), while BV2 microglial cells were subjected to the same LPS-stimulated conditions and treated with AECF (50–400 μg/mL) (**C**) or uracil (10–80 μM) (**D**); protein expression levels of iNOS and COX-2 were analyzed by Western blotting, with β-actin used as a loading control, and densitometric analysis was performed to quantify band intensities, with relative expression levels normalized to β-actin. Data are presented as mean ± SD (*n* ≥ 3), and statistical significance is indicated as * *p* < 0.05, ** *p* < 0.01, and *** *p* < 0.001 versus LPS-treated control. White bars indicate untreated control cells; pink bars indicate LPS-stimulated cells; orange bars represent AECF-treated groups; and green bars represent uracil-treated groups.

To evaluate the impact of AECF and uracil on the expression of inducible nitric oxide synthase (iNOS) and cyclooxygenase-2 (COX-2) proteins, RAW264.7 and BV2 microglia cells were incubated with AECF and uracil for 2 h and then stimulated with LPS for 24 h. The results showed that AECF and uracil significantly inhibited the LPS-induced expression of iNOS in both cell types (Figure 5). Additionally, these treatments clearly reduced COX-2 protein expression in BV2 microglial cells, whereas only a minimal and non-significant change was observed in RAW264.7 macrophages.

### 2.5. Inhibitory Effect of AECF and Uracil on NF-κB Translocation in RAW264.7 and BV2 Microglia Cells

**Figure 6:**
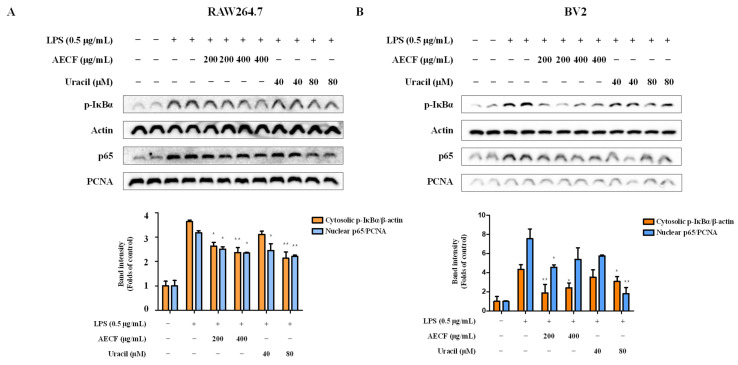

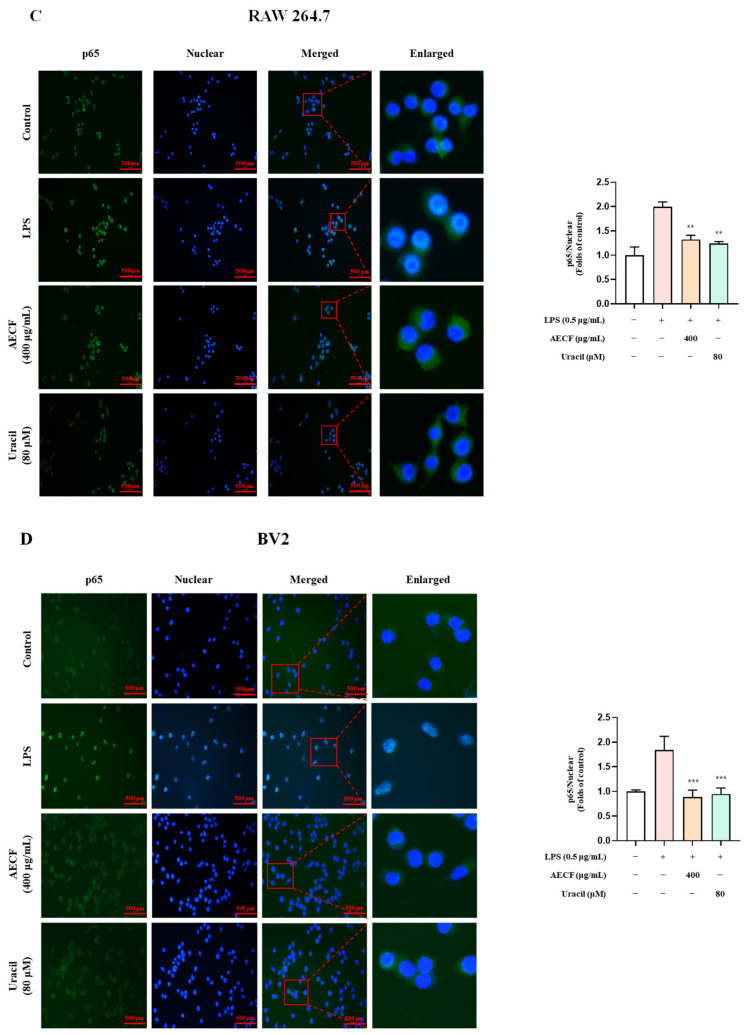
Western blot and immunofluorescence analysis of NF-κB signaling activation and p65 nuclear translocation in RAW264.7 and BV2 microglia cells treated with AECF or uracil were performed, in which RAW264.7 macrophages and BV2 microglial cells were stimulated with LPS (0.5 μg/mL) and treated with AECF (200–400 μg/mL) or uracil (40–80 μM); protein levels of phosphorylated IκBα (p-IκBα), cytosolic NF-κB p65, and nuclear NF-κB p65 were assessed by Western blotting (**A**,**B**), using β-actin and PCNA as loading controls. Band intensities were quantified and normalized, as shown in the accompanying graphs. In contrast, immunofluorescence staining was conducted to visualize NF-κB p65 and nuclei (DAPI) in RAW264.7 (**C**) and BV2 (**D**) cells, with merged images indicating that LPS-induced nuclear translocation of p65 was reduced by AECF or uracil treatment; scale bars are indicated. p65/nuclear fluorescence intensity ratios were quantified using ImageJ (imageJ 1.54g). Data are presented as mean ± SD (*n* = 3), and statistical significance is indicated as * *p* < 0.05, ** *p* < 0.01, and *** *p* < 0.001 versus LPS-treated control. White bars indicate untreated control cells; pink bars indicate LPS-stimulated cells; orange bars represent AECF-treated groups; and green bars represent uracil-treated groups.

To explore the mechanisms through which AECF and uracil suppress inflammation, we examined their effects on NF-κB (p65) targets. First, we determined that AECF and uracil inhibit the phosphorylation of IκB and prevent the LPS-induced nuclear translocation of p65 in RAW264.7 and BV2 microglia cells. This suggests that AECF and uracil block the activation of NF-κB signaling triggered by LPS (Figure 6A,B). To further validate this mechanism, we conducted immunofluorescence analysis to assess the cellular localization of p65. The results showed that, in RAW 264.7 and BV2 microglia cells treated with LPS, p65 translocated from the cytoplasm to the nucleus, thereby promoting inflammation. However, treatment with AECF and uracil effectively inhibited the nuclear translocation of p65 (Figure 6C,D). Together, the results of our study suggest that AECF and uracil have a modulating effect on NF-κB signaling. Notably, AECF and uracil decreased p65 nuclear localization and p-IκBα protein levels in a concentration-dependent manner in RAW264.7 cells. In BV2 microglial cells, however, AECF at 400 μg/mL produced a slightly less pronounced decrease in both p65 nuclear localization and p-IκBα levels than AECF at 200 μg/mL. These findings suggest that excessively high concentrations of AECF may induce cellular stress or other high-dose effects in BV2 cells, which could partially counteract its inhibitory action on NF-κB.

## 3. Discussion

Neurodegenerative diseases, such as Alzheimer’s disease, are closely linked to chronic neuroinflammation, primarily mediated by the activation of microglia within the central nervous system (CNS) [35,36,37]. Under resting conditions, microglia maintain neuronal homeostasis; however, upon stimulation with inflammatory agents such as LPS, they polarize into the M1 phenotype and secrete large amounts of pro-inflammatory mediators, including NO, tumor necrosis factor-alpha (TNF-α), and IL-6 [38]. These inflammatory responses are largely controlled by intracellular signaling cascades, such as the NF-κB pathway.

RAW 264.7, a murine macrophage-like cell line, is derived from a tumor in a BALB/c mouse induced with the Abelson murine leukemia virus. These cells possess the ability to perform pinocytosis and phagocytosis and are responsive to various stimuli, such as LPS, which induces NO production and enhances phagocytic activity. They are widely used as models for studying macrophage functions, including phagocytosis, cytokine production, and inflammatory responses [39]. The BV2 cell line is widely used as an in vitro model to investigate neuroinflammation and neurodegenerative disorders. Derived from primary microglial cultures of C57BL/6 mice, these cells were immortalized by infection with a J2 retrovirus harboring the v-raf and v-myc oncogenes. BV2 microglia cells provide a stable and reproducible alternative to primary microglia, as they retain key functional and morphological features, including the expression of typical microglial surface markers (e.g., MAC1 and MAC2). Additionally, they exhibit immune responsiveness by secreting pro-inflammatory cytokines in response to stimuli such as LPS [40,41,42].

The NF-κB pathway is one of the most well-characterized inflammatory response mechanisms. Normally, NF-κB remains inactive in the cytoplasm, bound to its inhibitory protein, IκB. However, in response to LPS, IκB is phosphorylated and degraded, allowing NF-κB to translocate into the nucleus and promote the transcription of pro-inflammatory genes, including iNOS and COX-2 [43,44,45,46,47]. Overexpression of these proteins contributes significantly to the inflammatory microenvironment and has been implicated in neuronal damage. RAW264.7 macrophages and BV2 microglia are widely used as in vitro models to study inflammation. LPS-induced activation of these cells is commonly employed to mimic inflammatory conditions and evaluate the anti-inflammatory efficacy of candidate compounds [48,49,50,51]. However, the nuclear translocation of NF-κB p65 appeared to be enhanced at the highest concentrations. Combined with the cell viability assessment using the MTT assay, at the highest concentration of AECF, it may trigger a stress response. In this study, uracil was isolated from *C. fragile*. To the best of our knowledge, this is the first report demonstrating that naturally occurring uracil isolated from *C. fragile* exerts anti-inflammatory and anti-neuroinflammatory effects in RAW264.7 macrophages and BV2 microglial cells. Previous studies have mainly focused on synthetic uracil derivatives or evaluated uracil in other disease models, and no study has investigated the anti-inflammatory mechanism of uracil itself in these two cell lines. Our findings, therefore, extend the pharmacological profile of both uracil and AECF by identifying NF-κB as a common molecular target in microglial and macrophage models. In the HPLC chromatogram of the AECF, uracil was detected as a major peak (Figure 1B), and quantitative analysis revealed a content of 2.290 ± 0.039 mg/g (0.2%). The ultimate goal of our research is to develop pharmaceuticals or functional health foods utilizing AECF. Therefore, we approached this study with the objective of identifying marker compounds using HPLC, which is the most widely accessible and standardized analytical instrument. These findings suggest that uracil can be used as a reliable marker for the standardization of *C. fragile*. Accordingly, the anti-inflammatory and anti-neuroinflammatory slight deviations from the clear y effects of AECF and uracil were simultaneously evaluated in LPS-stimulated RAW264.7 and BV2 microglia cells. We first assessed the cytotoxicity of AECF and uracil in both cell types and confirmed that the extract exhibited no cytotoxicity at concentrations up to 400 μg/mL, while uracil showed no cytotoxic effects at concentrations below 80 μM (Figure 2A,C). The effects of the AECF and uracil on NO production (Figure 2B,D), pro-inflammatory cytokine release (Figure 3), and the expression of iNOS and COX-2 (Figure 5) were evaluated. IL-10 is widely recognized as an anti-inflammatory cytokine that limits excessive inflammatory responses. In macrophages, IL-10 is often induced following LPS stimulation as a delayed autoregulatory mechanism, and additional immunomodulatory cues can further augment IL-10 production [27,28,29]. Consistent with this concept, our Figure 4A shows that AECF and uracil further enhance IL-10 production under LPS challenge, suggesting that these treatments may reinforce anti-inflammatory feedback signaling rather than merely suppressing pro-inflammatory mediators. In microglia, however, IL-10 responses to LPS are heterogeneous across studies, with reports describing reduced IL-10 levels as well as unchanged IL-10 secretion depending on experimental context [30,31,32,33]. In Figure 4B, LPS significantly decreased IL-10, while AECF/uracil co-treatment showed an upward trend toward recovery without statistical significance versus the LPS group in BV2 microglia cells. This pattern suggests a potential normalizing effect on an LPS-disrupted anti-inflammatory milieu, although the magnitude of restoration appears modest under our current conditions. Both AECF and uracil decreased the production of inflammatory cytokines and expression of iNOS and COX-2 proteins. Furthermore, the nuclear translocation of NF-κB was assessed using immunofluorescence staining to determine whether AECF and uracil exert their effects by modulating NF-κB signaling (Figure 6).

Nevertheless, the present study has several limitations. First, previous studies have reported that co-treatment with LPS and anti-inflammatory agents in RAW264.7 macrophages and BV2 microglial cells leads to increased IL-10 production, which has been used as a marker reflecting anti-inflammatory responses [27,31,52]. Consistent with these reports, the present study demonstrated that co-treatment with LPS and AECF or uracil resulted in a significant increase in IL-10 production in RAW264.7 cells. In contrast, in BV2 microglial cells, although an increasing trend in IL-10 production was observed under the same treatment conditions, the changes did not reach statistical significance. Therefore, given that IL-10 induction is often time-dependent and can vary by cell type and experimental timing, future studies should include time-course analyses and upstream pathway assessments (e.g., IL-10 mRNA expression and IL-10-associated signaling such as STAT3/SOCS3) to clarify whether AECF/uracil actively induce IL-10 or primarily normalize suppressed IL-10 under LPS challenge in BV2 microglia cells. Second, the present work is limited to in vitro experiments using RAW264.7 macrophages and BV2 microglia cells. In vivo studies employing relevant models of neuroinflammation or neurodegenerative diseases will be required to confirm the protective effects of AECF and uracil at the organismal level. Third, in BV2 microglial cells, the inhibitory effect of AECF on NF-κB signaling tended to plateau or slightly attenuate at the highest concentration tested, suggesting that potential high-dose effects should be carefully evaluated in future mechanistic and preclinical studies.

In summary, our findings demonstrate that an aqueous extract of *Codium fragile* (AECF) and its low-molecular-weight component uracil significantly attenuate LPS-induced inflammatory responses in RAW264.7 macrophages and BV2 microglial cells. Both AECF and uracil inhibited the production of NO, IL-6, and TNF-α while enhancing IL-10 production, downregulated iNOS and COX-2 expression, and inhibited NF-κB p65 nuclear translocation in these cell models. To the best of our knowledge, this is the first report showing that naturally occurring uracil isolated from *C. fragile* exerts anti-inflammatory and anti-neuroinflammatory effects via modulation of the NF-κB pathway in both macrophage and microglial cells. These results not only extend the pharmacological profile of *C. fragile* but also highlight uracil as a useful chemical marker and a potential bioactive contributor to the anti-neuroinflammatory properties of AECF, supporting its further development as a candidate material for functional foods or pharmaceutical applications targeting neuroinflammatory conditions.

## 4. Materials and Methods

### 4.1. Chemicals and Reagents

The reagents used for cell culture, including Roswell Park Memorial Institute (RPMI) 1640, phosphate-buffered saline (PBS), fetal bovine serum (FBS), penicillin, and trypsin-EDTA, were purchased from Gibco BRL Co. (Grand Island, NY, USA). The 3-[4,5-dimethylthiazol-2-yl]-2,5-diphenyltetrazolium bromide (MTT) reagent for cytotoxicity assays was obtained from Sigma Chemical Co. (St. Louis, MO, USA). The dimethyl sulfoxide (DMSO) used in the cytotoxicity experiments was purchased from Sigma Chemical Co. (St. Louis, MO, USA). The IL-6, TNF-α, and IL-10 Mouse ELISA Kits were acquired from BioLegend Co. (San Diego, CA, USA). All other chemicals were purchased from Sigma-Aldrich (St. Louis, MO, USA). Primary antibodies, including anti-iNOS, anti-COX-2, anti-p-IκB, anti-p65, anti-PCNA, anti-Actin, and anti-proliferating cell nuclear antigen (PCNA), were purchased from Cell Signaling Technology (Danvers, MA, USA). Horseradish peroxidase (HRP)-conjugated anti-rabbit and anti-mouse secondary antibodies were purchased from Millipore (Billerica, MA, USA).

### 4.2. Preparation of the Aqueous Extracts of C. fragile (AECF)

*Codium fragile* was purchased from Wando (Republic of Korea). It was washed several times with tap water to remove salt and then dried. Thereafter, the dried sample was extracted with water at 90 °C for 4 h and concentrated and spray-dried. Subsequently, the extracted powder was stored at 4 °C.

### 4.3. Seaweed Materials, Extracts, and Isolation

The AECF was dissolved in water and sequentially fractionated with dichloromethane, ethyl acetate, and n-butanol, yielding four fractions. High-performance liquid chromatography (HPLC) was used to identify the major marker compound in the n-butanol fraction. This fraction was dissolved in methanol and subjected to open-column chromatography using a YMC*GEL ODS-A (YMC Co., Ltd., Kyoto, Japan) column (3.5 × 21 cm), eluted with a gradient of 10–100% methanol in water, resulting in subfractions (CG-B10–100). The HPLC analysis indicated that CG-B10 (479 mg) was suitable for further purification. It was dissolved in 20% methanol and purified using Sephadex LH-20 column chromatography with 20% methanol. The CG-B10-S5 fraction was found to contain the highest concentrations of the target compounds. Preparative HPLC was then performed using a YL-9100 HPLC system (YoungLin, Anyang, Republic of Korea) equipped with a Phenomenex Kintex 5 µm C18 100 Å column (150 × 21.2 mm I.D., 5 µm, Phenomenex, Torrance, CA, USA) at a flow rate of 5 mL/min. The mobile phase consisted of 0.1% formic acid in water (A) and methanol (B), with a gradient of 2% MeOH (0–10 min) to 100% MeOH (10–20 min). The peak at 9.2 min was collected and concentrated to yield 3.5 mg of CG-B10-S5-1. The compound CG-B10-S5-1 was analyzed using a JEOL JNM ECP-400 NMR spectrometer (JEOL Ltd., Tokyo, Japan) in DMSO-d_6_ (600 µL). ^1^H-NMR (DMSO-d_6_, 400 MHz): δ 10.85 (1H, brs, OH-3), 7.41 (1H, d, J = 7.3 Hz, H-6), and 5.44 (1H, d, J = 7.8 Hz, H-5); ^13^C-NMR (DMSO-d_6_, 100 MHz): 164.4 (C-4), 151.6 (C-2), 142.4 (C-6), and 100.2 (C-5). High-resolution mass spectrometry (HR-MS) was performed at the Korea Polar Research Institute, yielding the following results: positive mode: 113.0341 *m*/*z* [M + H]^+^ (calculated for C_4_H_5_N_2_O_2_, 113.0351); negative mode: 111.0202 *m*/*z* [M-H]^−^ (calculated for C_4_H_3_N_2_O_2_, 111.0195). Based on the NMR and MS data, the compound was identified as uracil (Figures S2–S5) [53].

### 4.4. HPLC Analysis Methods

HPLC analysis was performed using a SHIMADZU system equipped with a UV-Vis detector (Shimadzu, Kyoto, Japan). The separation was carried out on a Kintex 5 µm C18 100 Å (Phenomenex, Torrance, CA, USA) column (4.6 × 250 mm, 5 µm). The mobile phase consisted of water containing 0.1% formic acid (A) and acetonitrile (B), with the following gradient elution program: 1% B for 5 min, increasing to 20% B over 13 min (5–18 min), followed by an increase to 100% B within 2 min (18–20 min), which was maintained for 2 min (20–22 min). The composition was then decreased to 1% B over 2 min (22–24 min) and then maintained at 1% B for 6 min (24–30 min) for re-equilibration. The flow rate was set at 0.7 mL/min, and detection was performed at 254 nm. The column temperature was maintained at 30 °C, and the injection volume was 20 µL (Tables S1 and S2).

### 4.5. Cell Culture and Viability Assays

RAW264.7 was purchased from American Type Culture Collection (ATCC, Manassas, VA, USA). The BV2 microglia cells were donated by Prof. Youn-Chul Kim (College of Pharmacy, Wonkwang University, Iksan, Republic of Korea). RAW264.7 and BV2 microglia cells were cultured in RPMI medium supplemented with 10% (*v*/*v*) fetal bovine serum (FBS) at 37 °C in a humidified 5% CO_2_ atmosphere. 3-(4,5-dimethylthiazol-2-yl)-2,5-diphenyltetrazolium bromide (MTT) reagent (0.5 mg/mL) was added to the wells of the cell culture plate and incubated at 37 °C for 1 h to assess cell viability. DMSO was added to dissolve formazan. Absorbance of each sample was measured at 540 nm using an ELISA microplate reader (Molecular Devices, San Jose, CA, USA).

### 4.6. Measurement of Nitrite Generation

To evaluate the production of nitrite, mouse microglia BV2 microglia cells and mouse macrophages RAW264.7 were treated with LPS and AECF or uracil. The AECF and uracil were dissolved in DMSO and diluted in cell culture medium prior to treatment. The final concentration of DMSO did not exceed 0.1% (*v*/*v*). All treatments were administered via direct addition to the cell culture wells [54]. After incubation for 24 h, the supernatant (100 μL) was mixed with the Griess Reagent A and Griess Reagent B (100 μL) and then reacted. Measurements were performed at a wavelength of 570 nm using an ELISA microplate reader (Molecular Devices, San Jose, CA, USA).

### 4.7. Assays for IL-6, TNF-α, and IL-10

The culture medium was collected to determine IL-6, TNF-α, and IL-10 levels using the appropriate cytokine ELISA kits (BioLegend, San Diego, CA, USA) as described by the manufacturer. Briefly, RAW264.7 and BV2 microglia cells (5 ×10^5^ cells/well) were seeded and incubated in 48-well culture plates. AECF and uracil were treated, and the supernatant of each well in which an inflammatory reaction occurred was analyzed at a wavelength of 450 nm.

### 4.8. Western Blot Analysis

The pelleted BV2 and RAW264.7 cells were washed with PBS and lysed in RIPA buffer. Equal amounts of protein were quantified using a protein assay dye reagent concentrate obtained from Bio-Rad Laboratories (#5000006; Hercules, CA, USA), mixed in sample loading buffer, and separated by SDS-PAGE. The separated proteins were then transferred onto nitrocellulose membranes. Nonspecific binding to the membrane was blocked by incubation with skim milk. The membranes were incubated with primary antibodies (all of which were used at a ratio of 1:1000) at 4 °C overnight. After washing thrice with TBST, the membranes were incubated with a horseradish peroxidase-conjugated secondary antibody (1:5000 dilution) for 1 h at room temperature.

### 4.9. NF-κB Localization and Immunofluorescence

We investigated the localization of NF-κB in RAW264.7 and BV2 microglia cells, which were seeded on Lab-Tek II chamber slides. The cells were incubated with uracil (80 μM) or AECF (400 μg/mL) for 3 h, stimulated with LPS (0.5 μg/mL) for 15 min, and then fixed in formalin and permeabilized with cold acetone. The cells were probed with p65 antibodies and incubated with a secondary antibody labeled with Alexa Fluor 488 (Invitrogen, Carlsbad, CA, USA). Nuclei were visualized by incubation with 0.5 mg/mL of 4′,6-diamidino-2-phenylindole (DAPI) for 5 min, followed by washing with PBS five times. The cells were then incubated with VectaShield (Vector Laboratories, Burlingame, CA, USA). Stained cells were visualized and photographed using a Provis AX70 fluorescence microscope (Olympus Optical Co., Tokyo, Japan).

### 4.10. Statistical Analysis

All data were acquired from three independent experiments and expressed as mean ± SD. Statistical analyses were performed using GraphPad Prism software version 5.01 (GraphPad Software Inc., San Diego, CA, USA). The mean difference was determined with one-way analysis of variance (ANOVA), followed by Tukey’s multiple comparison test, and statistical significance was defined as *p* < 0.05.

## 5. Conclusions

This study investigated the anti-inflammatory and anti-neuroinflammatory effects of uracil, a natural compound isolated from the marine-derived alga *C. fragile* and its aqueous extract. Uracil was identified as the major low-molecular-weight component and chemical marker of the aqueous extract, and both uracil and the extract were found to suppress inflammation in a concentration-dependent manner in macrophages and microglia. This anti-inflammatory activity was confirmed through the inhibition of the NF-κB signaling pathways. In conclusion, these results suggest that *C. fragile* has potential for development as an anti-inflammatory therapeutic agent, an anti-dementia drug, or a functional health food.

## Data Availability

The data presented in this study are available upon request from the corresponding authors.

## Supplementary Materials

The following supporting information can be downloaded at: https://www.mdpi.com/article/10.3390/md24010038/s1, Figure S1: Calibration curve of Uracil standard solution; Figure S2: ^1^H NMR spectrum of uracil; Figure S3: ^13^C NMR spectrum of uracil; Figure S4: HR-ESIMS (positive) spectrum of uracil; Figure S5: HR-ESIMS (negative) spectrum of uracil; Table S1: HPLC conditions for the analysis of *C. fragile* extract; Table S2: HPLC analysis of *C. fragile* extract was performed using solvent A (distilled water containing 0.1% formic acid) and solvent B (acetonitrile) under a gradient elution system.

## Abbreviations

The following abbreviations are used in this manuscript:

AECF	aqueous extract of *Codium fragile*
HPLC	high performance liquid chromatography
NMR	nuclear magnetic resonance
MS	mass spectrometry
DMSO	dimethyl sulfoxide
NO	nitric oxide
NF-κB	nuclear factor-kappa B
MAPK	mitogen-activated protein kinase
IL	interleukin
iNOS	inducible nitric oxide synthase
COX	cyclooxygenase
LPS	lipopolysaccharide
ELISA	enzyme-linked immunosorbent assay
TNF	tumor necrosis factor
DAPI	4′,6-diamidino-2-phenylindole
