# Supplementary material for: Anti-Neuroinflammatory Effects of a Representative Low-Molecular-Weight Component Isolated from Codium fragile Through Inhibition of the NF-κB Pathway in Microglia and Macrophage Cells
Source: Mar Drugs. 2026 Jan 13;24(1):38. doi: 10.3390/md24010038 (PMC12843343; doi:10.3390/md24010038)
Supplement: Supplementary file 1 [file marinedrugs-24-00038-s001.zip › marinedrugs-3981274-supplementary.pdf]

Supplementary data

Article

Anti-neuroinflammatory effects of a major component from *Codium fragile* through inhibition of the NF-κB pathway in microglia and macrophage cells

Gyoyoung Lee[1,†], Yezhi Jin[1,†] Seul Ah Lee[2], Sook-Young Lee[3], Hwan Lee[1], Zisheng Nan[1], Chi-Su Yoon[4,*], and Dong-Sung Lee[1,*]

[1] Research Institute of Pharmaceutical Sciences (RIPS), College of Pharmacy, Chosun University, Gwangju 61452, Republic of Korea; dlrydud612@naver.com (G.L), yezhi102@gmail.com (Y.J) ghksdldi123@hanmail.net (H.L.) zisheng1125@gmail.com (Z.N)
[2] Department of Oral Biochemistry, College of Dentistry, Chosun University, Gwangju 61452, Republic of Korea; seulah21@naver.com (S.A.L.)
[3] Marine Healthcare Research and Evaluation Center, Chosun University, Wando 59146, Republic of Korea; seedbank@chosun.ac.kr (S.-Y.L.)
[4] Institute of Pharmaceutical Research and Development, College of Pharmacy, Wonkwang University, Iksan 54538, Republic of Korea
* Correspondences: ycs91@wku.ac.kr (C.-S.Y); Tel.: +82-63-850-6823(C.-S.Y); dslee2771@chosun.ac.kr (D.-S.L.); Tel.: +82-62-230-6386(D.-S.L.)
† These authors contributed equally to this work.

Contents

Table S1. HPLC conditions for the analysis of *C. fragile* extract.

Table S2. HPLC analysis of *C. fragile* extract was performed using solvent A (distilled water containing 0.1% formic acid) and solvent B (acetonitrile) under a gradient elution system.

Figure S1. Calibration curve of Uracil standard solution.

Figure S2. ^{1}H NMR spectrum of uracil.

Figure S3. ^{13}C NMR spectrum of uracil.

Figure S4. HR-ESIMS (positive) spectrum of uracil.

Figure S5. HR-ESIMS (negative) spectrum of uracil.

Table S1. HPLC conditions for the analysis of *C. fragile* extract.

Parameter	Condition
HPLC system	SHIMADZU(Nexera, SOLVENT DELIVERY MODULELC-40D)
Column	Kintex5 μm C18 100 Å (Phenomenex)
Detectot	UV 254 nm
Flow rate	0.7 mL/min
Column temperature	30℃
Injection volume	20 μL

Table S2. HPLC analysis of *C. fragile* extract was performed using solvent A (distilled water containing 0.1% formic acid) and solvent B (acetonitrile) under a gradient elution system.

Time (min)	(A)	(B)
Initial	99	1
5	99	1
18	80	20
20	0	100
22	0	100
24	99	1
30	99	1

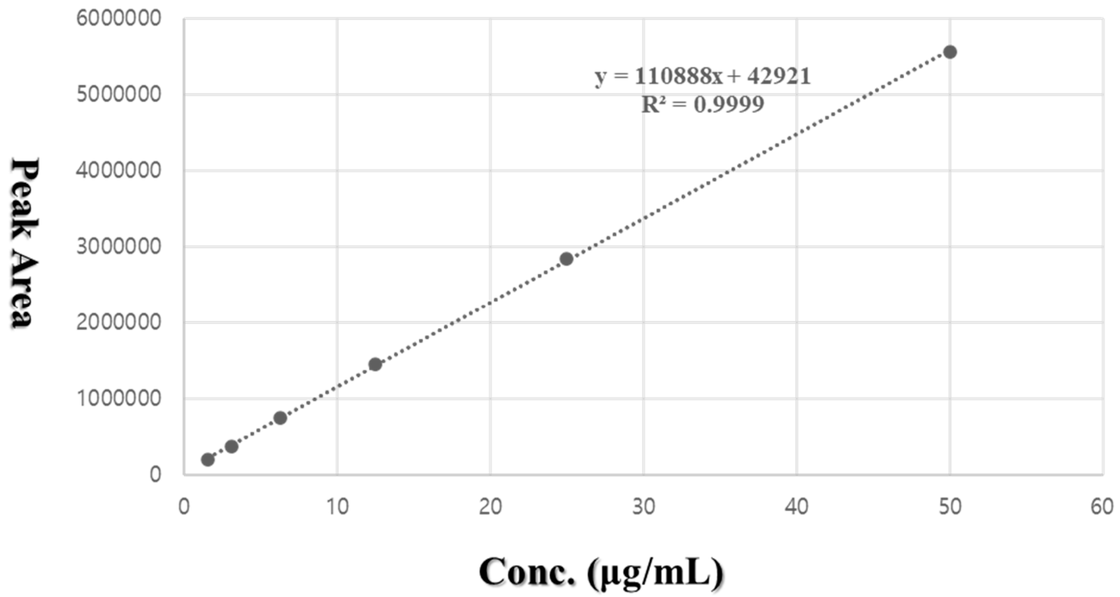

Figure S1. Calibration curve of Uracil standard solution.

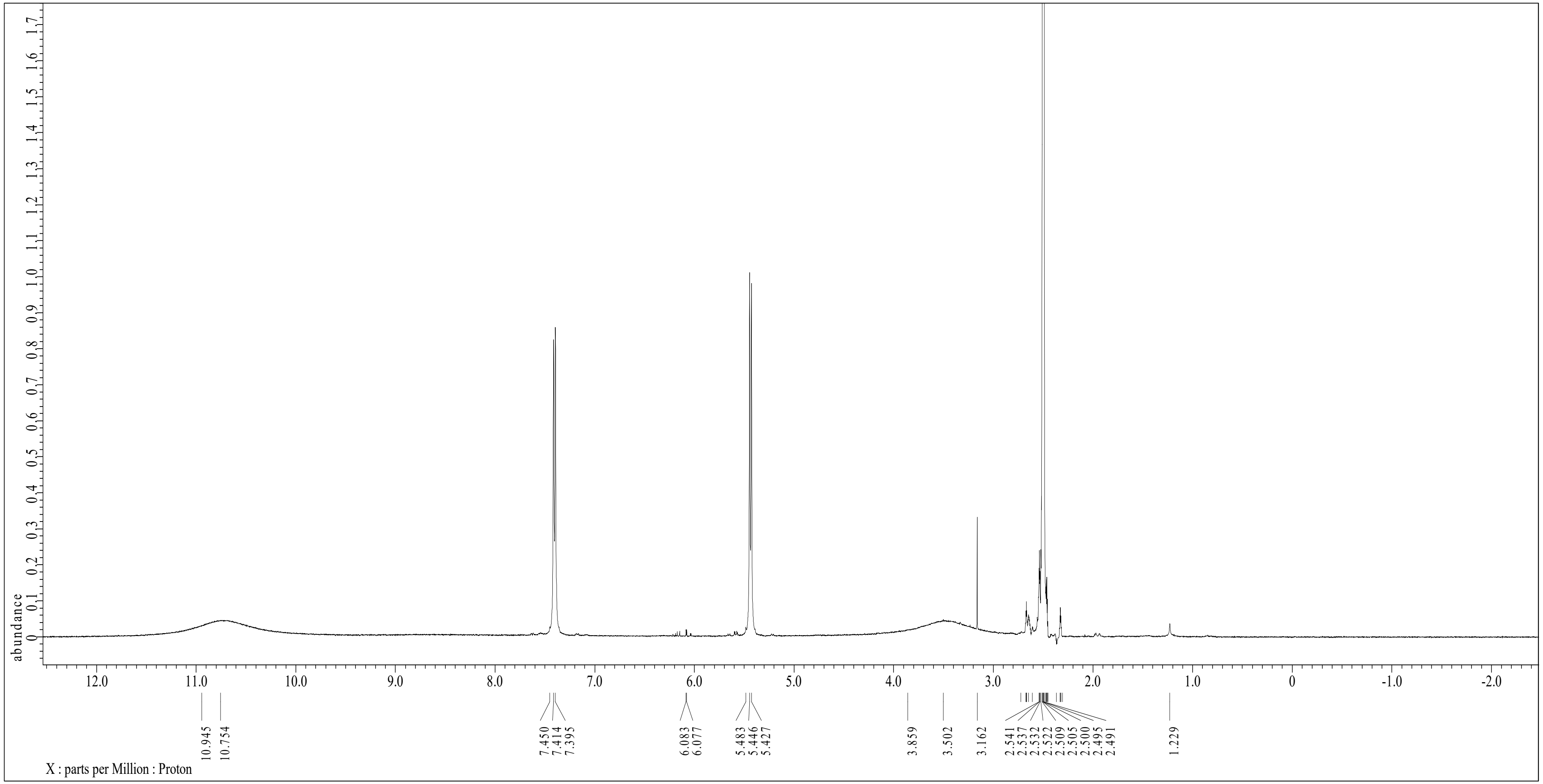

Figure S2. ^{1}H NMR spectrum of uracil.

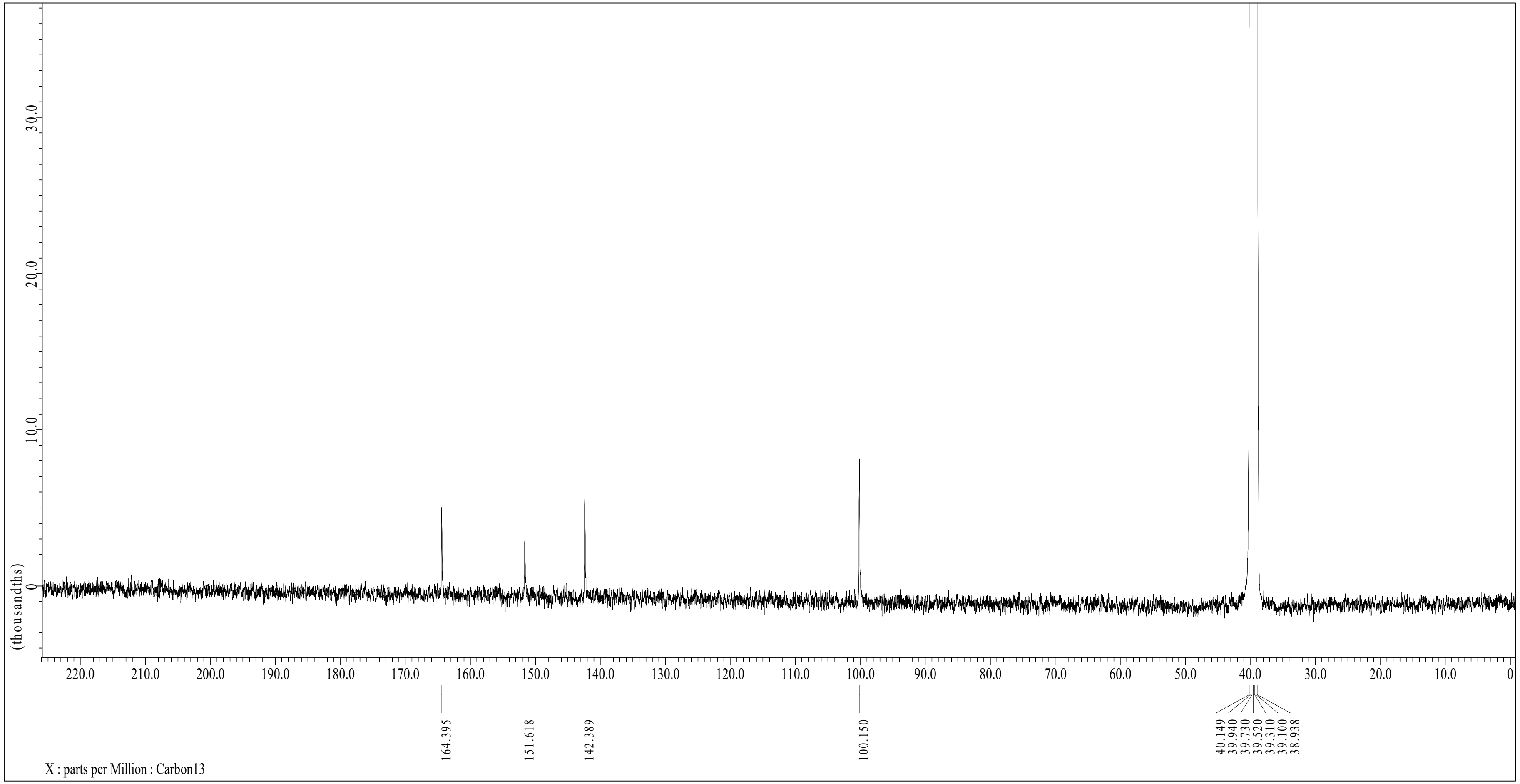

Figure S3. ^{13}C NMR spectrum of uracil.

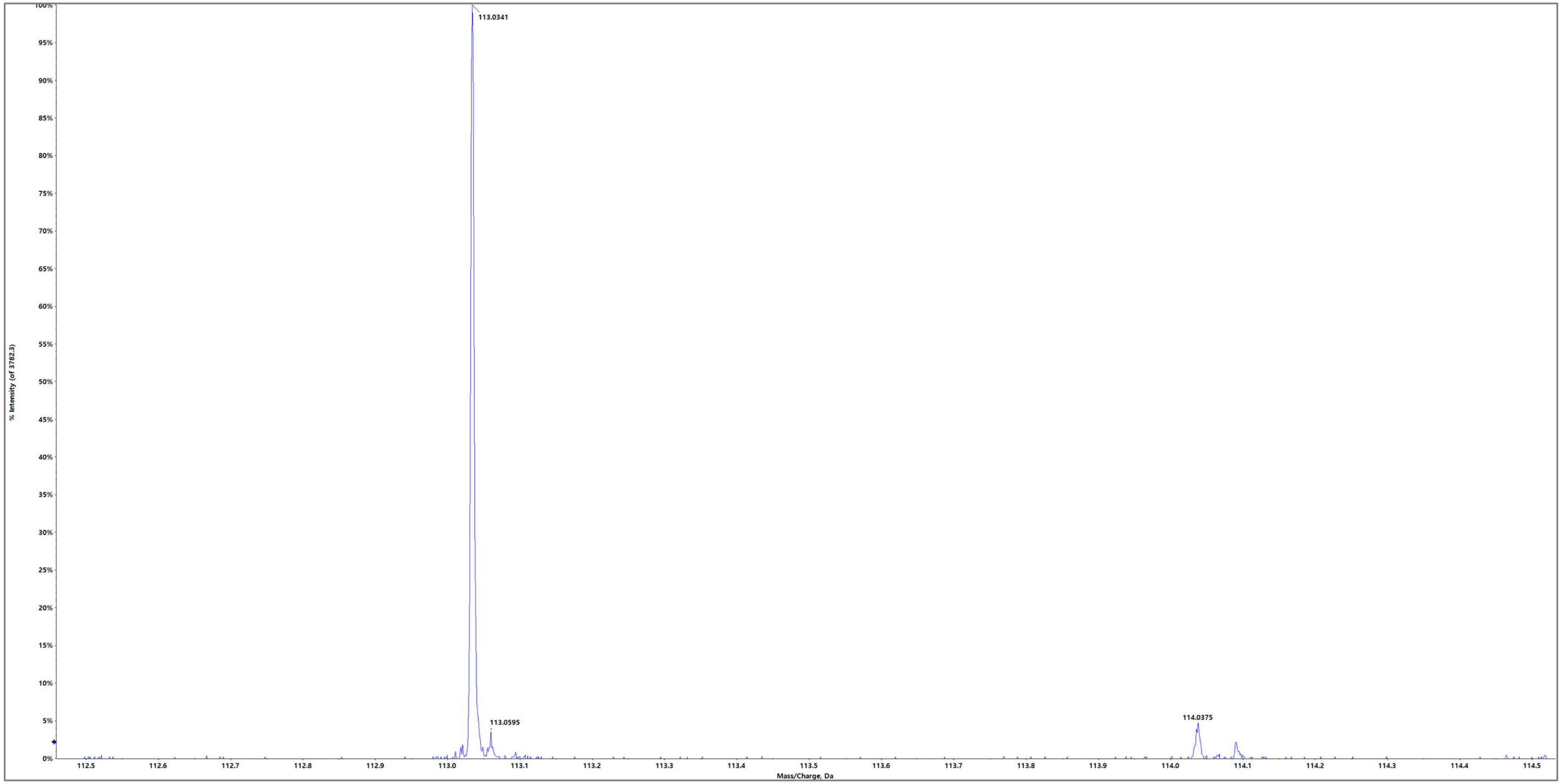

Figure S4. HR-ESIMS (positive) spectrum of uracil.

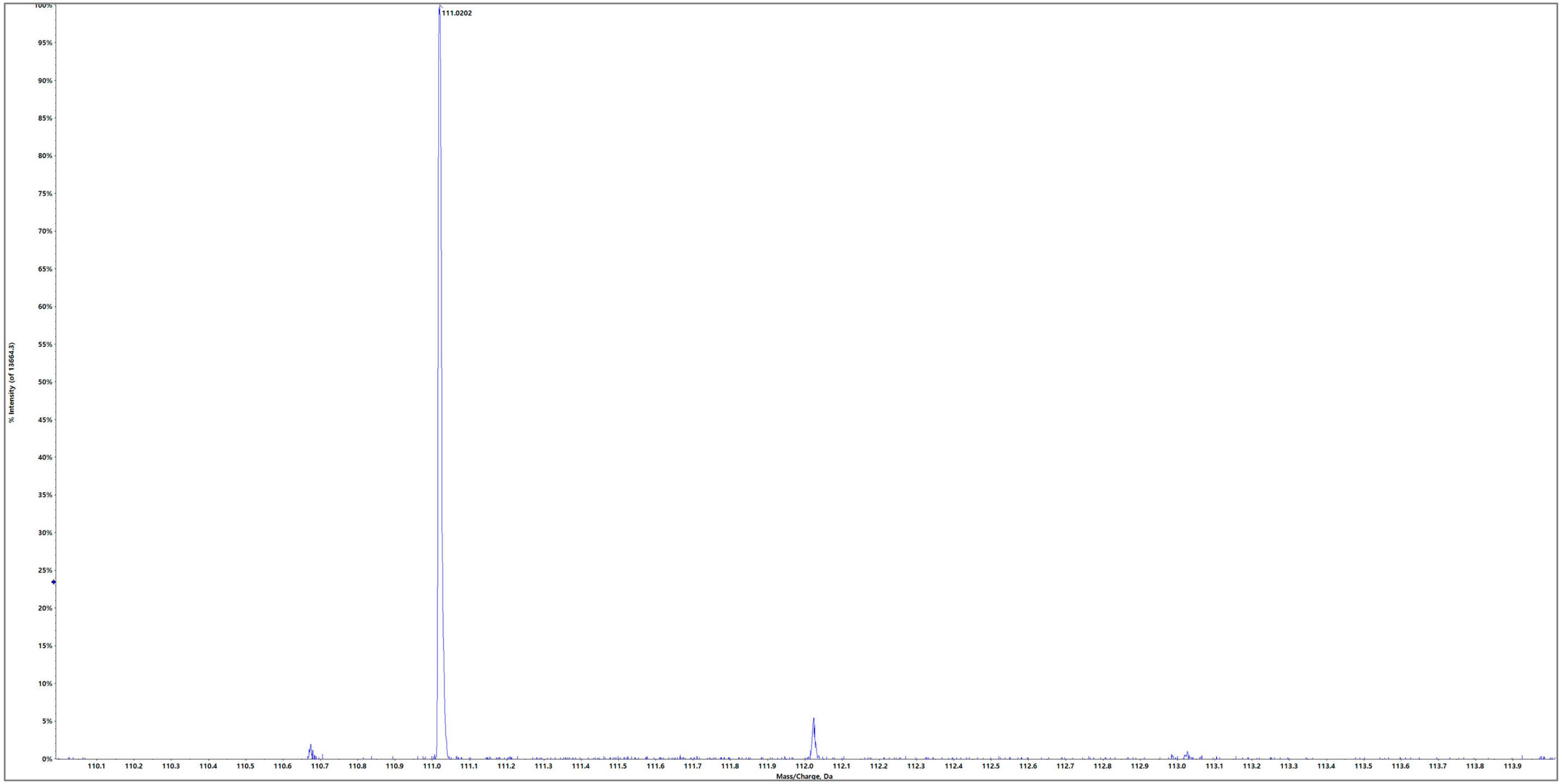

Figure S5. HR-ESIMS (negative) spectrum of uracil.
